# Analysis of the Setup Uncertainty and Margin of the Daily ExacTrac 6D Image Guide System for Patients with Brain Tumors

**DOI:** 10.1371/journal.pone.0151709

**Published:** 2016-03-28

**Authors:** Se An Oh, Ji Woon Yea, Min Kyu Kang, Jae Won Park, Sung Kyu Kim

**Affiliations:** 1 Department of Radiation Oncology, Yeungnam University Medical Center, Daegu, Korea; 2 Department of Radiation Oncology, Yeungnam University College of Medicine, Daegu, Korea; 3 Department of Radiation Oncology, Kyungpook National University School of Medicine, Daegu, Korea; Taipei Medical University, TAIWAN

## Abstract

This study evaluated the setup uncertainties for brain sites when using BrainLAB’s ExacTrac X-ray 6D system for daily pretreatment to determine the optimal planning target volume (PTV) margin. Between August 2012 and April 2015, 28 patients with brain tumors were treated by daily image-guided radiotherapy using the BrainLAB ExacTrac 6D image guidance system of the Novalis-Tx linear accelerator. DUON^TM^ (Orfit Industries, Wijnegem, Belgium) masks were used to fix the head. The radiotherapy was fractionated into 27–33 treatments. In total, 844 image verifications were performed for 28 patients and used for the analysis. The setup corrections along with the systematic and random errors were analyzed for six degrees of freedom in the translational (lateral, longitudinal, and vertical) and rotational (pitch, roll, and yaw) dimensions. Optimal PTV margins were calculated based on van Herk et al.’s [margin recipe = 2.5∑ + 0.7σ - 3 mm] and Stroom et al.’s [margin recipe = 2∑ + 0.7σ] formulas. The systematic errors (∑) were 0.72, 1.57, and 0.97 mm in the lateral, longitudinal, and vertical translational dimensions, respectively, and 0.72°, 0.87°, and 0.83° in the pitch, roll, and yaw rotational dimensions, respectively. The random errors (*σ*) were 0.31, 0.46, and 0.54 mm in the lateral, longitudinal, and vertical rotational dimensions, respectively, and 0.28°, 0.24°, and 0.31° in the pitch, roll, and yaw rotational dimensions, respectively. According to van Herk et al.’s and Stroom et al.’s recipes, the recommended lateral PTV margins were 0.97 and 1.66 mm, respectively; the longitudinal margins were 1.26 and 3.47 mm, respectively; and the vertical margins were 0.21 and 2.31 mm, respectively. Therefore, daily setup verifications using the BrainLAB ExacTrac 6D image guide system are very useful for evaluating the setup uncertainties and determining the setup margin.

## Introduction

With the advent of new technology such as intensity modulated radiotherapy (IMRT) and volumetric modulated radiotherapy (VMAT), a radiotherapy treatment planning system (RTPS) can maximize the dose to the tumor while minimizing the dose to the normal organs [1]. Therefore, using a planning target volume (PTV) with a small margin can achieve a steep dose gradient and homogeneous dose distributions between the tumor and planning organ at risk volume (PRV).

The PTV expands the clinical target volume (CTV) by an appropriate margin. The internal margin (IM) accounts for the variation in the size or shape of the tumor, and the setup margin (SM) accounts for uncertainties in the position of the patient [2].

One reason that the expansion of the PTV margin from the CTV can be reduced has been the development of image guide systems. Recently, image guide systems such as ExacTrac (BrainLAB, Feldkirchen, Germany) and cone beam computed tomography (CBCT) (Varian Medical System, CA, USA) have played an important role in improving the accuracy of patient positioning and target localization for radiotherapy [3–9].

Many studies have tried to quantify the margin between the CTV and PTV by using the electronic portal imaging device (EPID) [10, 11], on-board imager (OBI) (Varian Medical System, CA, USA) [12], and CBCT [13–17] for translational variations in the lateral (*x*), longitudinal (*z*), and vertical (*y*) dimensions of the brain sites.

Recently, Infusino et al. [18] reported the setup uncertainties and optimal margin of stereotactic radiation therapy (SRT) (30 Gy/3 fractions) for rotational variations in the pitch, roll, and yaw dimensions at the brain sites with the X-Ray ExacTrac 6D system (BrainLAB, Feldkirchen, Germany) having six degrees of freedom (DOF).

This study analyzed setup images for the multi-fractionation (54 Gy/27 fractions to 59.4 Gy/33 fractions) of 28 brain tumor patients with 844 X-ray image registrations. The objective of this study was to evaluate the patient setup uncertainties for daily pretreatment using ExacTrac 6D images and determine the optimal PTV margin for brain sites receiving multi-fractionation (27–33 fractions).

## Materials and Methods

### Ethics statement

**Fig 1:**
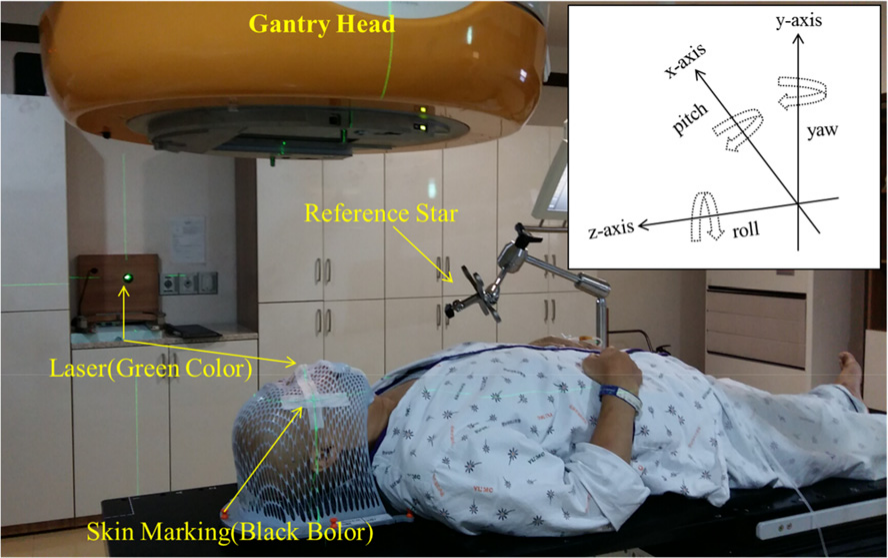
Image of the Patient Setup. DUON^TM^ immobilization masks were used to fix the head in the translational (lateral, longitudinal, and vertical) and rotational (pitch, roll, and yaw) dimensions.

This study was approved by the Institutional Review Board (IRB) of the Yeungnam University Medical Center (YUMC 2015-12-006), and patient consent was specially waived under the approval of the YUMC IRB because the patient data were investigated anonymously. The individual pictured in Fig 1 of this manuscript has given written informed consent (as outlined in the PLOS consent form) to publish these case details.

### Patient selection

Twenty-eight patients with brain tumors treated consecutively between November 2012 and April 2015 were included in this study. The ExacTrac 6D (BrainLAB, Feldkirchen, Germany) image guidance system of the Novalis-Tx (BrainLAB, Feldkirchen, Germany) linear accelerator was used for the daily image guided radiotherapy (IGRT). In total, the scale of the treatment involved 27–33 fractionations. A total of 844 image verifications were performed for the 28 patients and used for the analysis. Table 1 presents the characteristics of the patients included in the study. The ages of the 17 female and 11 male patients were 19–77 years with an average age of 54.5 years. The average PTV was 262.7 cm^3^ with a range of 26.7–569.5 cm^3^.

**Table 1:** Characteristics of the Patients in the Study.

Patient No.	Gender	Age	Tumor site	Technique used for treatment	Prescription dose/fractionations	PTV[cm^3^]
1	F	31	Rt temporal lobe	IMRT	54 Gy/30	89.0
2	F	30	Temporal lobe	IMRT	54 Gy/30	145.1
3	M	63	Temporal lobe	IMRT	54 Gy/30	143.0
4	M	56	Lt temporal-parietal lobe	IMRT	59.4 Gy/33	450.4
5	F	41	Lt temporal-occipital lobe	IMRT	54 Gy/30	209.6
6	M	74	Occipital lobe	IMRT	60 Gy/30	371.7
7	F	19	Temporal-parietal lobe	VMAT	54 Gy/30	123.1
8	M	55	Rt frontal-parietal lobe	CRT	60 Gy/30	496.1
9	M	44	Lt frontal lobe	IMRT	60 Gy/30	99.2
10	M	47	Lt temporal-parietal-occipital lobe	IMRT	59.4 Gy/33	546.1
11	F	51	Lt temporal-parietal-occipital lobe	IMRT	59.4 Gy/33	463.5
12	F	74	Lt frontal lobe	IMRT	60 Gy/30	373.2
13	M	32	Lt Parietal lobe	VMAT	60 Gy/30	242.0
14	F	77	Frontal-temporal lobe	VMAT	54 Gy/30	120.3
15	F	65	Lt frontal-temporal lobe	IMRT	60 Gy/30	332.7
16	F	56	Lt temporal-parietal-occipital lobe	IMRT	60 Gy/30	401.7
17	F	69	Lt temporal lobe	IMRT	59.4 Gy/33	26.7
18	M	64	Rt temporal lobe	IMRT	60 Gy/30	130.5
19	F	45	Rt frontal lobe	IMRT	60 Gy/30	232.6
20	F	40	Lt frontal lobe	IMRT	54 Gy/27	65.2
21	F	75	Lt frontal lobe	IMRT	60 Gy/30	69.2
22	F	54	Lt frontal-parietal lobe	IMRT	54 Gy/27	189.2
23	M	58	Rt temporal-parietal lobe	IMRT	60 Gy/30	327.4
24	F	75	Rt temporal lobe	IMRT	60 Gy/30	224.7
25	F	66	Rt temporal-parietal-occipital lobe	IMRT	60 Gy/30	382.3
26	M	64	Rt temporal lobe	IMRT	60 Gy/30	130.5
27	F	72	Lt temporal-parietal-occipital lobe	IMRT	60 Gy/30	401.2
28	M	30	Rt frontal-temporal-occipital lobe	IMRT	50.4 Gy/28	569.5

The PTV was contoured by the initial simulation computed tomography (CT).

Abbreviations: IMRT = Intensity modulated radiotherapy; VMAT = Volumetric modulated radiotherapy; CRT = Conventional radiotherapy; PTV = Planning target volume

### Immobilization and simulation

DUON^TM^ (Orfit Industries, Wijnegem, Belgium) masks were used as a frameless immobilization device to fix the head. Fig 1 presents an image of the patient setup using the DUON^TM^ masks with a BrainLAB infrared (IR) reflective reference star for the brain treatment. All patients were scanned with a Brilliance Big Bore CT simulator (Philips Inc., Cleveland, OH) with a thickness of 2 mm.

### Treatment planning and delivery technique

A Novalis Tx (Varian Medical System, CA, USA and BrainLAB, Feldkirchen, Germany) linear accelerator machine with HD-120 MLC was used for this study. The Novalis Tx was equipped with an MV electronic portal imaging device (EPID) and kV on-board-imager (OBI) and kV ExacTrac 6D image for the image guide system. Only the kV ExacTrac 6D image guide system was used for the results provided in this paper.

As shown in Fig 1, the treatment techniques used in this study were IMRT, VMAT, and conventional radiotherapy (CRT). Twent*y-*four patients were treated with IMRT, three patients were treated with VMAT, and one patient was treated with CRT. Eclipse 8.6 (Varian Medical System, Palo Alto, CA, USA) was used as the treatment planning system (TPS). Radiation was delivered to the tumor at a dose rate of 600 MU/min with a photon energy of 6 MV.

### Image registration and setup protocol

**Fig 2:**
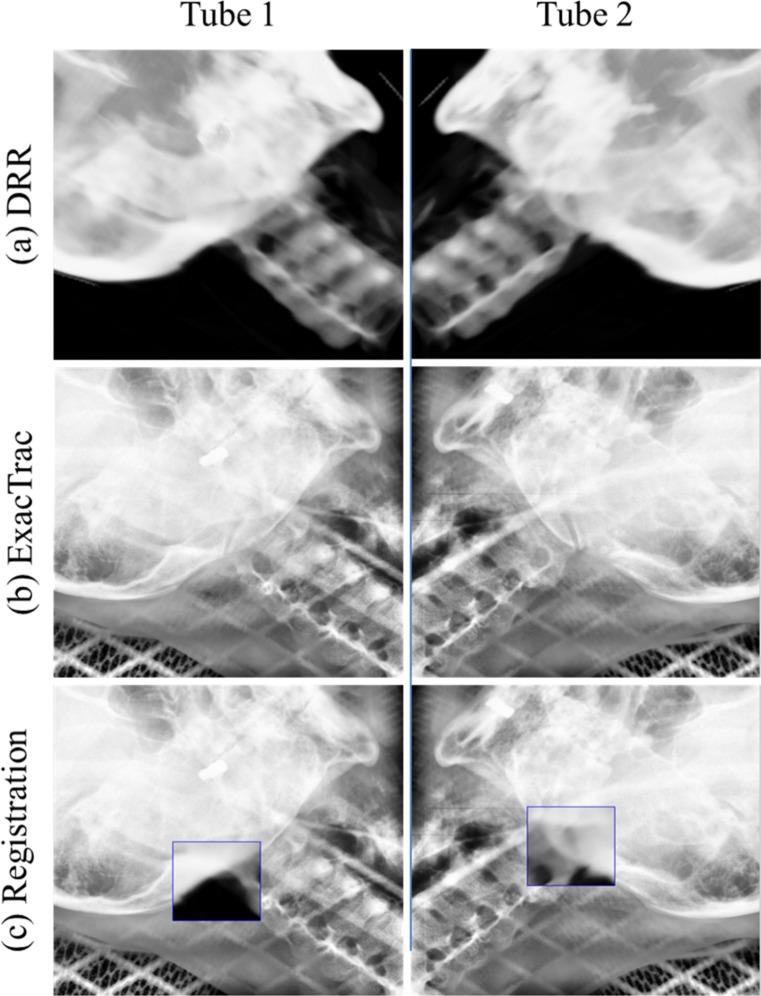
Image Registration Using kV Tubes 1 and 2 with BrainLAB ExacTrac for Patient #2. (a) DRR image, (b) ExacTrac image, and (c) image registration.

All 28 patients underwent daily ExacTrac 6D (BrainLAB AG, Feldkirchen, Germany) setup imaging using the two floor-mounted kV X-ray tubes. Fig 2 shows the image registration using the kV tubes 1 and tube 2 with BrainLAB ExacTrac for patient #2. Fig 2(A) shows a digitally reconstructed radiograph (DRR) from the CT simulation image, and Fig 2(B) was obtained with the ExacTrac system. Fig 2(C) shows the image registration between the CT simulation and ExacTrac image for 6 DOF in the translational (lateral, longitudinal, and vertical) and rotational (pitch, roll, and yaw) dimensions. The criterion for image registration between the CT simulation image and ExacTrac image was bony anatomy matching. All treatments were verified by image registration before each treatment fraction. The institute’s setup protocol for the brain IMRT was first matched by marking the skin on the mask in the treatment room. Second, the patient’s setup verification images were obtained with the BrainLAB ExacTrac system. Third, image registration between the CT simulation image and ExacTrac image was performed automatically by the BrainLAB 6D Fusion algorithms. Setup corrections for the six DOF in the translational (lateral, longitudinal, and vertical) and rotational (pitch, roll, and yaw) dimensions were automatically applied to the BrainLAB robotic couch system.

We recorded the setup errors to determine the systematic and random errors from imaging when the patient was in the final image guidance-based corrected position.

### Analysis of the setup variations for the systematic and random errors

The formula proposed by van Herk et al. [19] was used to analyze the setup errors for the random (σ) and systematic (∑) errors in the patient setup correction.

Remeijer et al. [20] defined σ and ∑ for the setup in detail as follows:

$$N=\sum_{p=1}^{P}F_{p}$$ (1)

$$M=\frac{1}{N}\sum_{p=1}^{P}\sum_{f=1}^{F_{p}}x_{pf}$$ (2)

$$\sigma_{p}=\sqrt{\frac{1}{F_{p}-1}\sum_{f=1}^{F_{p}}{\left(x_{pf}-m_{p}\right)}^{2}}$$ (3)

$$m_{p}=\sum_{f=1}^{F_{p}}\frac{x_{pf}}{F_{p}}$$ (4)

$$\sigma =\sqrt{\frac{1}{N-P}\sum_{p=1}^{P}(F_{p}-1)\cdot \sigma_{p}^{2}}=\sqrt{\frac{1}{N-P}\sum_{p=1}^{P}\sum_{f=1}^{F_{p}}{\left(x_{pf}-m_{p}\right)}^{2}}$$ (5)

$$\sum =\sqrt{\frac{P}{N(P-1)}\sum_{p=1}^{P}F_{p}{\left(m_{p}-M\right)}^{2}}$$ (6)

where *P* is the total number of patients, *F*_p_ is the measured fraction for each patient *p*, *N* is the total number of measured fractions, *x*_pf_ is the measured displacement of the patient *p* during the fraction *f* along the *x*-axis, *M* is the overall mean of all measurements, *m*_p_ is the patient average, *σ*_p_ is the standard deviation (SD) of the random errors for the single patient *p*, *σ* is the average SD of the random errors, and *∑* is the SD of the systematic errors.

### Calculation of the PTV margin from the CTV

The margin recipes of Stroom et al. [21] and Van Herk et al. [22] were used to calculate the PTV margin from the CTV. Stroom et al. assumed a 95% dose to 99% of the CTV on average based on tests of realistic plans:

Stroom et al.’s formula=2∑+0.7σ (7)

Van Herk et al. assumed a Monte Carlo-based test of 1% TCP loss due to geometric errors for the prostate. Their formula is defined as follows:

Van Herk et al.’s formula=2.5∑+0.7σ−3mm (8)

## Results and Discussion

**Fig 3:**
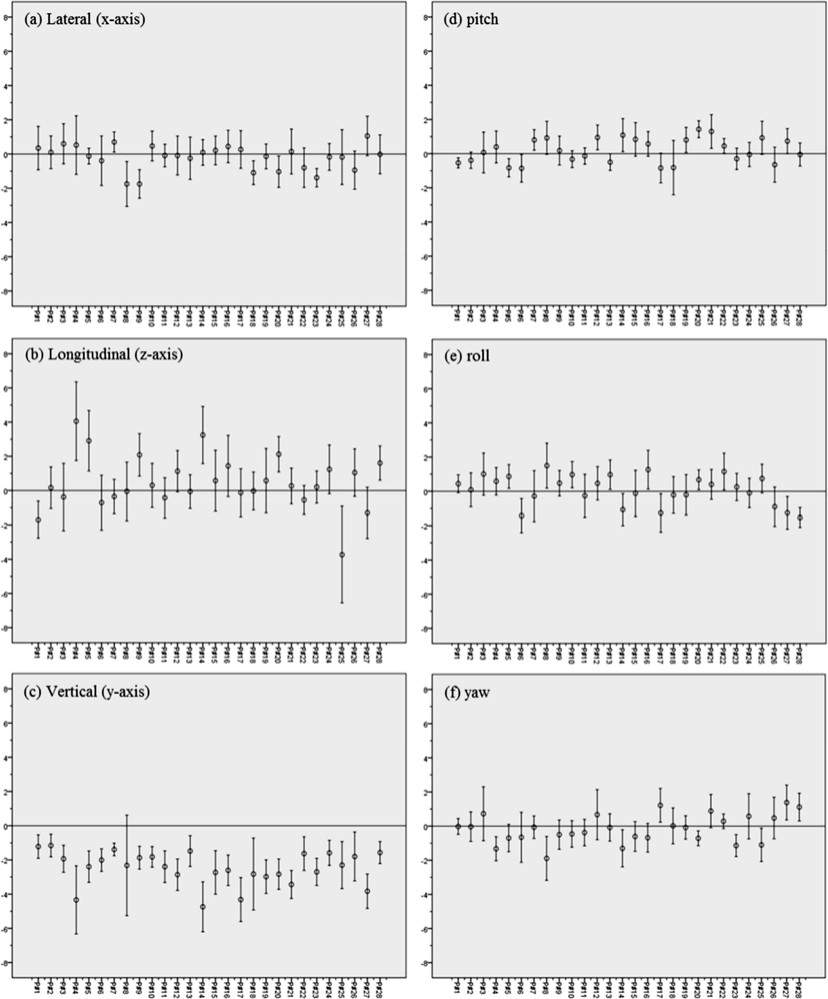
Inter-fractional Setup Variations (mean ± SD) for the 28 Patients. Setup errors in the (a) lateral (*x-*axis, left to right), (b) longitudinal (*z-*axis, superior to inferior), (c) vertical (*y-*axis, anterior to posterior), (d) pitch, (e) roll, and (f) yaw dimensions.

**Fig 4:**
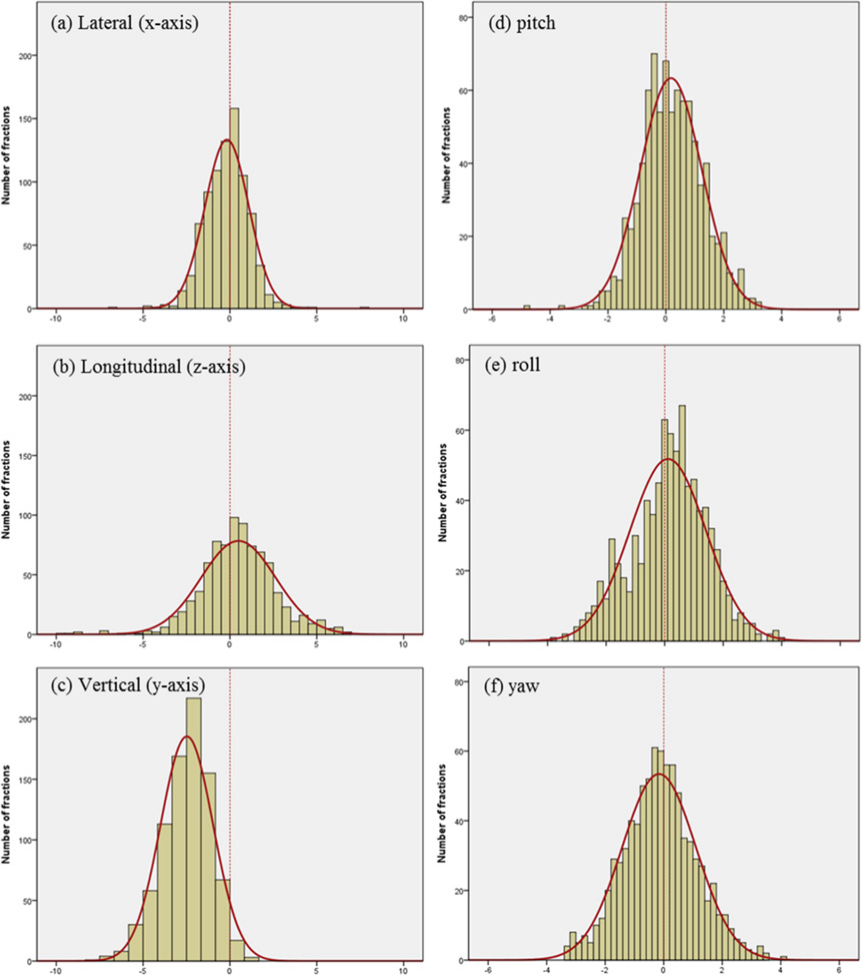
Histograms and Normalized Curves for the Translation and Rotational Variations. Setup errors in the (a) lateral (*x-*axis, left to right), (b) longitudinal (*z-*axis, superior to inferior), (c) vertical (*y-*axis, anterior to posterior), (d) pitch, (e) roll, and (f) yaw dimensions.

Fig 3 indicates the inter-fractional setup variations with the mean and the standard deviation (SD) in the lateral, longitudinal, vertical, pitch, roll, and yaw dimensions for the 28 patients. Fig 4 shows the histograms and normalized curves for the translation and rotational variations. For most dimensions, the average systematic error was close to zero. In the vertical dimensions, however, the average systematic error shifted to the negative directions.

Table 2 lists the systematic (Σ) and random (*σ*) errors in the translational (lateral (*x-*axis), longitudinal (*z-*axis), vertical (*y-*axis)) and rotational (pitch (*x-*axis), roll (*z-*axis), and yaw (*y-*axis)) dimensions. For the systematic errors, the lateral, longitudinal, vertical, pitch, roll, and yaw dimensions had mean values of -0.19 mm, 0.48 mm, -2.47 mm, 0.18°, 0.12°, and -0.16°, respectively, and standard deviations of 0.72 mm, 1.57 mm, 0.97 mm, 0.72°, 0.87°, and 0.83, respectively. For the random errors, the mean values were 1.01 mm, 1.43 mm, 1.06 mm, 0.76°, 0.97°, and 0.92°, respectively, and the standard deviations were 0.31 mm, 0.46 mm, 0.54 mm, 0.28°, 0.24°, and 0.31°, respectively.

**Table 2:** Systematic (Σ) and Random (*σ*) Errors in the Translational and Rotational Dimensions.

	Systematic error (Σ)		Random error (*σ*)	
	Mean	SD	Mean	SD
Translational				
Lateral (*x-*axis) [mm]	-0.19	0.72	1.01	0.31
Longitudinal (*z-*axis) [mm]	0.48	1.57	1.43	0.46
Vertical (*y-*axis) [mm]	-2.47	0.97	1.06	0.54
Rotational				
Pitch (*x-*axis) [°]	0.18	0.72	0.76	0.28
Roll (*z-*axis) [°]	0.12	0.87	0.97	0.24
Yaw (*y-*axis) [°]	-0.16	0.83	0.92	0.31

Infusino et al. [18] evaluated the setup uncertainties for brain sites with the ExacTrac X-Ray 6D system when performing stereotactic radiotherapy with a dose schedule of 30 Gy/3 fractions on 15 patients. They measured the systematic errors as less than 2.0 mm in all directions. In the present study, the systematic error in the vertical direction was -2.47 mm compared to the 1.89 mm of Infusino et al. Their random errors were smaller for all patients at 0.1–0.3 mm compared to the random errors in the present study of 0.76–1.43 mm in all directions. Compared to the CTV-to-PTV margins of Infusino et al. [18] with the ExacTrac image tool, our residual setup errors were much smaller: -0.9 mm, -1.6 mm, -1.8°, -0.4°, and -1.8° for the lateral, vertical, pitch, roll, and yaw dimensions, respectively, with Stroom et al.’s formula [21]; and -0.2 mm, -2.1 mm, -0.5°, and -1.4° for the longitudinal, vertical, pitch, and yaw dimensions, respectively, with Van Herk et al.’s formula [22].

Shi et al. [8] reported a 2 mm setup uncertainty in translation and less than 0.25° uncertainty in rotation for 43 patients undergoing prostate IMRT treatment with the ExacTrac X-ray 6D system.

Hong et al. [12] investigated the setup errors for on-board imager (OBI) kV image verification with a BrainLAB thermoplastic head face mask consisting of a rear occipital mask, nasal bride, and two strips for cranial stereotactic radiosurgery (SRS) and stereotactic radiotherapy (SRT) of 42 patients with 57 brain lesions. The mean and SD of the couch shift were 0.0±0.9, 0.1±1.4, and 0.3 ±0.8 mm in the vertical, longitudinal, and lateral directions, respectively. In all directions, the mean value was close to 0. SRS masks of different types were used for strong fixation of the brain.

Oh et al. [13] evaluated the setup uncertainties for sites of the brain, head and neck, thorax and abdomen, and prostate of a daily CBCT image guide system with four DOF. The systematic errors in the lateral, longitudinal, vertical, and yaw dimensions were 1.1 mm, 1.1 mm, 1.1 mm, and 0.7°, respectively, and the random errors were 1.4 mm, 1.0 mm, 0.7 mm, and 0.7°, respectively. These setup uncertainties are comparable to the results in the present study for brain sites with the ExacTrac image guide system.

The dose of the image guidance is important. Linthout et al. [23] noted that the radiation dose per X-ray tube with the ExacTrac system is 0.5 mSv. For a single verification, the patient absorbs a dose of 1 mSv. This dose is low compared to cone beam computed tomography (CBCT), where the dose is 14.0 mSv.

Table 3 presents the CTV to PTV margins calculated according to the margin recipes proposed by Stroom et al. [21] and van Herk et al. [24] for the systematic (Σ) and random (*σ*) errors. In the results, the CTV to PTV margins in the lateral, longitudinal, vertical, pitch, roll, and yaw dimensions were 1.7 mm, 3.5 mm, 2.3 mm, 1.6°, 1.9°, and 1.9°, respectively, with Stroom et al.’s formula [21] and 1.0 mm, 1.3 mm, 0.2 mm, 1.0°, 0.7°, and 0.7°, respectively, with van Herk et al.’s formula [22].

Stroom et al.’s formula [21] was demonstrated to be accurate for a prostate, cervix, and lung cancer case with a CTV-to-PTV margin size ensuring at least a 95% dose to 99% of the CTV. Our results indicated that the dose coverage of CTV ensured at least a 95.02% dose to 99% of the CTV for brain sites in 28 patients. These results showed that Stroom et al.’s formula [21] is good for brain sites.

**Table 3:** Calculated CTV to PTV Margins Proposed by Stroom et al.’s [21] and van Herk et al.’s [22, 24] Formulas Based on the Systematic (Σ) and Random (*σ*) Errors.

Series	Image tool	Recipe	PTV margin					
			Translational			Rotational		
			Lateral	Longitudin	Vertical	Pitch	Roll	Yaw
			(mm)	al (mm)	(mm)	(°)	(°)	(°)
Infusino et al. [18]	ExacTrac	Stroom et al, 1999	2.6	3.4	3.9	3.4	2.3	3.7
		Van Herk et al, 2002	0.5	1.5	2.3	1.5	0.1	2.1
Oh et al. [13]	CBCT	Van Herk et al, 2000	3.73	3.45	3.24			
Zhou et al [16]	MVCT	Stroom et al, 1999	4.8	5.0	1.5			
Kataria et al. [17]	CBCT	Stroom et al, 1999	3.6	3.1	3.4			
		Van Herk et al, 2000	4.2	3.5	4.0			
Present study	ExacTrac	Stroom et al, 1999*^h^	1.7	3.5	2.3	1.6	1.9	1.9
		Van Herk et al, 2002*	1.0	1.3	0.2	1.0	0.7	0.7

^*^ Stroom et al.’s formula [21] = 2 ∑ + 0.7*σ*, Van Herk et al.’s formula [22] = 2.5 ∑ + 0.7*σ* − 3*mm*

Zhou et al. [16] reported the CTV to PTV margin for 15 brain sites with 25–35 fractions of megavoltage computed tomography (MVCT). Patients were fixed to a type-S^TM^ head extension board (CIVCO, Orange City, IA) with a thermoplastic facemask. By using Stroom et al.’s formula, Zhou et al. determined the margins to be 4.8, 5.0, and 1.5 mm in the lateral, longitudinal, and vertical dimensions, respectively.

Kataria et al. [17] evaluated the setup uncertainties by using a kv-CBCT image guide system for 15 brain patients. A daily image guide system such as kv-CBCT can be used as a tool to further reduce the PTV margin if daily online correction and analysis of the residual errors are performed.

**Fig 5:**
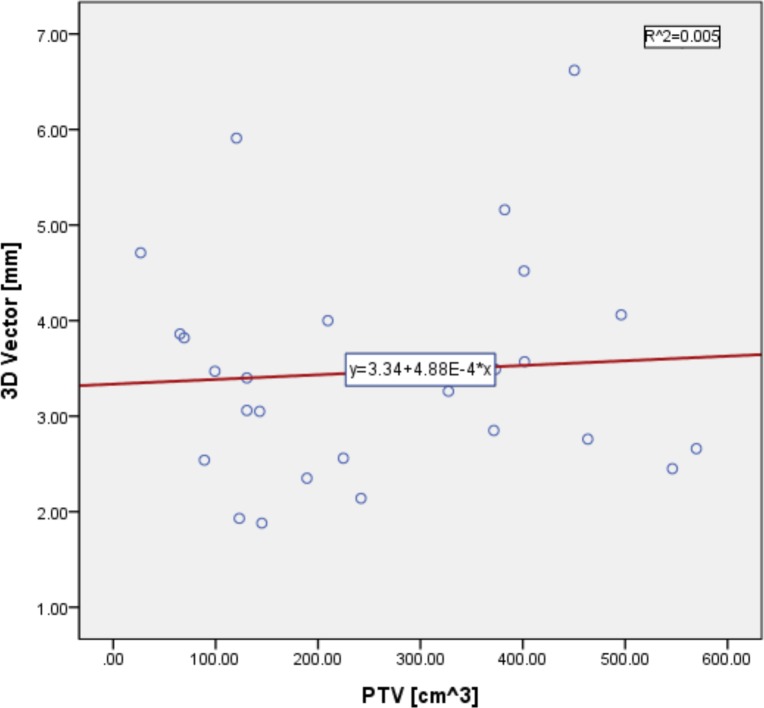
Spearman Correlation Analysis between the 3D Vector [mm] and PTV Volume [cm^3^]. *Spearman correlation coefficient *ρ* = -0.004; p value = 0.985; R^2^ = 0.005

The Spearman correlation was used to analyze the relationship between the 3D vector and PTV volume, as shown in Fig 5. The 3D vector can be calculated as follows:

$$3DVector=\sqrt{x^{2}+y^{2}+z^{2}}$$ (9)

where *x*, *y*, and *z* are the lateral, longitudinal, and vertical dimensions, respectively.

Because the Spearman correlation coefficient was -0.004, the linear correlation between the 3D vector and PTV volume can generally be neglected (p value = 0.985).

One limitation of our study is that we only considered bony rigid image registration when using the ExacTrac 6D fusion algorithms. If we considered the variation in size or shape of the internal organs from the deformable registration, the setup uncertainties and margins may also have varied in this study.

Another issue was the intra-fractional variation illustrating the internal organ displacement and the inter-fraction variation illustrating the setup error. Previously published papers have addressed the intra-fractional motion [15, 16, 21, 23]. Beltran et al. [15] reported inter- and intra-fractional positional uncertainties in pediatric radiotherapy patients with brain tumors (*n* = 83) and head and neck tumors (*n* = 17) when using megavoltage cone-beam CT. Image registration was performed before each treatment and after every other treatment. The offsets of the pretreatment image registration were used to calculate the inter-fractional setup uncertainties, and the offsets of the post treatment image registration were used to calculate the intra-fractional residual uncertainties. The residual uncertainties were 0.5, 0.5, and 0.5 mm in the lateral, longitudinal, and vertical translational dimensions, respectively, for the systematic errors (∑) and 0.9, 0.9, and 1.1 mm in the lateral, longitudinal, and vertical translational dimensions, respectively, for the random errors (σ).

Unfortunately, we did not consider the intra-fractional variations in the present study. In future work, we plan to address both inter- and intra-fractional variations.

## Conclusion

The present study evaluated the patient setup uncertainties for 28 brain sites when using BrainLAB ExacTrac for pretreatment. In our study, the CTV-to-PTV margins in the lateral, longitudinal, vertical, pitch, roll, and yaw dimensions were calculated as 1.7 mm, 3.5 mm, 2.3 mm, 1.6°, 1.9°, and 1.9°, respectively, with Stroom et al.’s formula and 1.0 mm, 1.3 mm, 0.2 mm, 1.0°, 0.7°, and 0.7°, respectively, with van Herk et al.’s formula, respectively, for the brain IMRT multi-fractional radiation treatment.

Thus, daily setup verifications that use the BrainLAB ExacTrac 6D image guide system are very useful for evaluating the setup uncertainties and determining the setup margin.
